# The Molecular Role of Immune Cells in Dilated Cardiomyopathy

**DOI:** 10.3390/medicina59071246

**Published:** 2023-07-05

**Authors:** Enping Wang, Ruofan Zhou, Tiange Li, Yimin Hua, Kaiyu Zhou, Yifei Li, Shuhua Luo, Qi An

**Affiliations:** 1 Department of Cardiovascular Surgery, West China Hospital, Sichuan University, Chengdu 610041, China; enpingwang77@gmail.com (E.W.); ruofanzhou99@gmail.com (R.Z.); litiange1999@foxmail.com (T.L.); 2 Key Laboratory of Birth Defects and Related Diseases of Women and Children of MOE, Department of Pediatrics, West China Second University Hospital, Sichuan University, Chengdu 610041, China; nathan_hua@163.com (Y.H.); kaiyuzhou313@163.com (K.Z.); drshuhualuo@gmail.com (S.L.)

**Keywords:** dilated cardiomyopathy, immune cells, cytokines, immunotherapy

## Abstract

Dilated cardiomyopathy (DCM) is a rare and severe condition characterized by chamber dilation and impaired contraction of the left ventricle. It constitutes a fundamental etiology for profound heart failure and abrupt cardiac demise, rendering it a prominent clinical indication for heart transplantation (HTx) among both adult and pediatric populations. DCM arises from various etiologies, including genetic variants, epigenetic disorders, infectious insults, autoimmune diseases, and cardiac conduction abnormalities. The maintenance of cardiac function involves two distinct types of immune cells: resident immune cells and recruited immune cells. Resident immune cells play a crucial role in establishing a harmonious microenvironment within the cardiac tissue. Nevertheless, in response to injury, cardiomyocytes initiate a cytokine cascade that attracts peripheral immune cells, thus perturbing this intricate equilibrium and actively participating in the initiation and pathological remodeling of dilated cardiomyopathy (DCM), particularly during the progression of myocardial fibrosis. Additionally, immune cells assume a pivotal role in orchestrating the inflammatory processes, which are intimately linked to the prognosis of DCM. Consequently, understanding the molecular role of various immune cells and their regulation mechanisms would provide an emerging era for managing DCM. In this review, we provide a summary of the most recent advancements in our understanding of the molecular mechanisms of immune cells in DCM. Additionally, we evaluate the effectiveness and limitations of immunotherapy approaches for the treatment of DCM, with the aim of optimizing future immunotherapeutic strategies for this condition.

## 1. Introduction

Dilated cardiomyopathy (DCM) is distinguished by the expansion of the left ventricle and impaired systolic function, regardless of atypical loading circumstances, such as hypertension or valvular heart disease, and significant coronary artery pathology [1]. Among the various etiologies of cardiomyopathy, DCM is the most prevalent worldwide. The prevalence of this disorder among the adult population is approximately one in 2500 individuals, whereas the annual incidence among children varies between 0 and 57 cases per 100,000, with a higher likelihood of occurrence in males than females [2,3]. While symptomatic heart failure is the typical presentation of DCM, it can also lead to arrhythmias, thromboembolism, or even remain asymptomatic. Furthermore, DCM is the leading cause of cardiac arrest and the primary indication for HTx [4,5,6]. Despite advancements in the management of dilated cardiomyopathy and heart failure, a definitive cure is still lacking. The rates of rehospitalization and mortality associated with DCM remain high, with a 10-year survival rate of only 60% [7,8]. Immune system disorders and inflammatory reactions are intrinsic to the pathogenesis of DCM. Various immune cells and the cytokines they secrete are implicated as potential causative factors and therapeutic targets for DCM. Therefore, a comprehensive understanding of the roles played by different immune cell populations in the development and progression of DCM may offer novel therapeutic avenues [9,10].

This review provides a comprehensive understanding of the intricate engagement of heterogeneous immune cell populations in the pathogenesis of DCM and particularly emphasizes the regulatory factors and signaling mechanisms that are closely associated with this process. Moreover, a critical assessment of the efficacy and limitations of immunotherapy strategies employed in the management of DCM is provided, offering valuable insights into the future directions of DCM therapeutics.

## 2. Etiology of DCM

The mutation of several genes encoding sarcomere and desmosome structural components will lead to the occurrence of familial genetic DCM. Acquired DCM is caused by exposure to toxins, pathogens, systemic endocrine disorders, autoimmune diseases, and other factors.

### 2.1. Genetic DCM

DCM is a prevailing familial genetic disorder, wherein a hereditary origin is evident in 20–30% of cases [11,12]. To date, more than 50 DCM-associated genes have been characterized, encompassing prominent candidates [13]. A total of 12 genes (BAG3, DES, DSP, FLNC, LMNA, MYH7, PLN, RBM20, SCN5A, TNNC1, TNNT2, and TTN) with strong and definitive classifications have a role in DCM that has been clearly demonstrated in the literature [PMID: 33947203]. Research conducted in genome-wide association studies (GWAS) and multi-trait analyses in 5,521 DCM participants with structurally normal hearts identified 13 loci associated with DCM, including FLNC, FHOD3, BAG3, ALPK3, TTN, and GATA4 [PMID: 33495596]. Notably, truncating mutations in the TTN gene prevail as the most common genetic variants observed in familial DCM, accounting for approximately 25% of cases [12]. The protein encoded by the TTN gene assumes a pivotal role in conferring elasticity and regulating sarcomere contraction. Nonetheless, the existence of genetic and phenotypic heterogeneity signifies ongoing uncertainty for numerous families. Furthermore, emerging research indicates that “acquired” DCM may potentially stem from underlying genetic variations, representing plausible etiological factors [14].

### 2.2. Acquired DCM

Myocardial injury, arising from various etiological factors such as infectious agents, autoimmune diseases, pharmaceutical agents, and other environmental triggers, elicits an inflammatory response characterized by cytokine activation and immune system involvement [15,16,17]. Ultimately, these cascading events contribute to left ventricular dysfunction and dilation. Notably, patients diagnosed with myocarditis frequently exhibit varying degrees of myocardial injury, which can progress to the development of dilated cardiomyopathy (DCM) [18]. Remarkably, research has demonstrated that even in cases of infection-negative myocarditis, the subsequent progression to DCM remains plausible [19]. Furthermore, cardiac toxicity induced by excessive alcohol consumption, cocaine use, chloroquine administration, psychotropic medications including Clozapine and Olanzapine, as well as certain anti-tumor drugs, can significantly impair myocardial function, leading to the acquisition of dilated cardiomyopathy [16,20].

## 3. Inflammatory Processes in DCM

Whether caused by genetic or environmental factors, myocardial damage can trigger an inflammatory response, recruiting immune cells to the heart to repair the myocardium. Histopathological examination of myocardial tissue from DCM patients commonly reveals evidence of inflammation and altered gene expression patterns associated with immune cell activation [1]. Both cardiac resident immune cells and recruited immune cells, including neutrophils, monocytes, macrophages, and mast cells, are poised to govern remodeling [PMID: 34320366]. Immune cells release cytokines, promoting extracellular matrix remodeling, collagen deposition, and fibrosis [PMID: 24577970]. Fibrosis is one of the characteristic pathological characteristics of Dilated cardiomyopathy [PMID: 23824828]. Notably, patients with DCM who present with heart failure accompanied by reduced systolic function (HFrEF) exhibit heightened levels of cellular adhesion molecules, pro-inflammatory cytokines, and macrophage-related proteins, all of which frequently correlate with an unfavorable prognosis [12]. Animal studies have further elucidated the direct impact of pro-inflammatory cytokines (such as IL-1, IL-2, IL-6, and tumor necrosis factor) on myocardial function, including their potential to impair contractility, induce left ventricular enlargement, and compromise endothelial function [12].

## 4. The Composition of Immune Cells in the Heart

**Figure 1:**
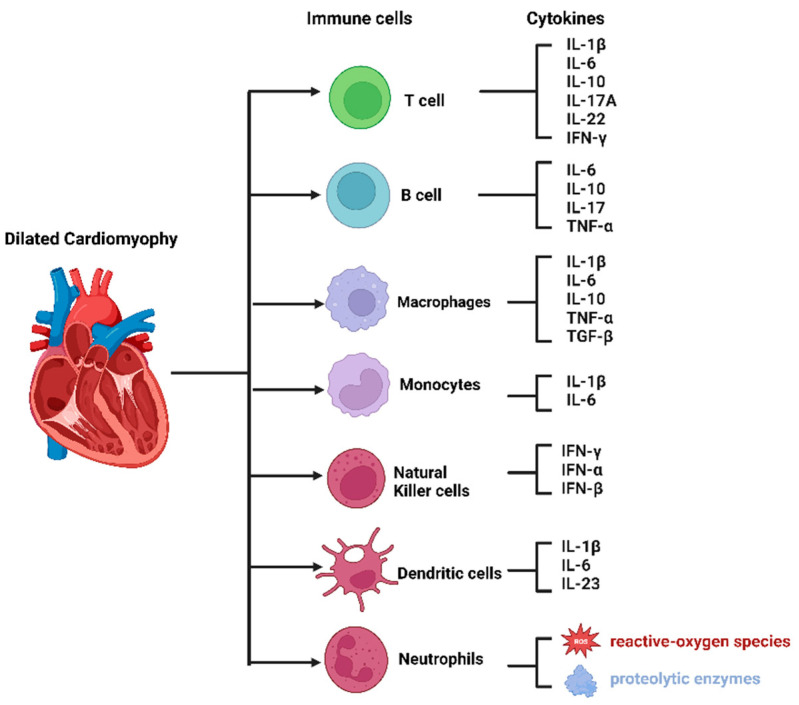
Immune cells and the main cytokines secreted in dilated cardiomyopathy.

The advent of single-cell RNA sequencing (scRNA-seq) technology has revolutionized our ability to accurately determine the proportions of immune cell populations residing within the cardiac milieu, including macrophages, dendritic cells, granulocytes, B and T cells, as well as natural killer (NK) cells [21,22] (Figure 1). Furthermore, scRNA-seq provides insights into the unique gene expression profiles exhibited by individual immune cell subsets, thereby enhancing our understanding of the transcriptional signatures underlying immune cell dynamics in the context of cardiac pathology. For example, Jung et al. [23] employed scRNA-seq to investigate a murine model of myocardial infarction, unraveling dynamic changes in macrophage populations over time, with these cardiac-resident macrophages displaying both inflammatory and protective transcriptional features. Similarly, Hua et al. [24] revealed scRNA-seq and revealed upregulation of the Hif1a gene, implicated in inflammation, in murine hearts during acute myocarditis, subsequently influencing the immune response of macrophages and T cells and exacerbating inflammatory processes. Moreover, they observed significantly elevated expression of Hif1a in individuals diagnosed with acute autoimmune myocarditis as compared to those with dilated cardiomyopathy and healthy controls [24]. For heart failure patients, Abplanalp et al. [25] showed increased FABP5 and signatures of Wnt signaling that could result in monocyte activation. In conclusion, single-cell RNA sequencing (scRNA-seq) has emerged as a potent investigative instrument in the realm of cardiovascular research, enabling the elucidation of cellular-level biological mechanisms, and is poised to contribute significantly to future scientific inquiries.

### 4.1. Innate Immune Cells

A diverse array of innate immune cells, including macrophages, dendritic cells (DCs), monocytes, and neutrophils, play pivotal roles in heart disease pathogenesis. Among these, cardiac macrophages, comprising approximately 7% of nonmyocytes within the adult mouse heart, represent integral constituents of the mononuclear phagocyte system [26]. Monocytes and macrophages are multifunctional, with characteristics such as antigen presentation, bactericidal activity, fibrosis, tissue healing, and regulating the immune system [27]. Under normal circumstances, neutrophils are not typically observed in healthy cardiac tissue. However, following heart tissue damage or trauma, neutrophils are recruited to the affected myocardium, instigating acute inflammation [28,29]. Dendritic cells predominantly function as antigen-presenting cells, facilitating the presentation of antigens to T cells within the context of cardiac tissue damage. Consequently, T cells undergo activation and mobilization to the site of cardiac injury [30].

### 4.2. Adaptive Immune Cells

The adaptive immune cell compartment comprises T cells and B cells, both of which play crucial roles in maintaining cardiac homeostasis and orchestrating responses to injury. Following cardiac injury, distinct subsets of pro-inflammatory and anti-inflammatory T cells are recruited to the damaged myocardium in response to antigen-presenting cells (APCs), such as dendritic cells [31,32]. Activated fibroblasts, macrophages, endothelial cells, and cardiomyocytes secrete chemotactic signals that directly affect T cells and/or indirectly influence them via APCs. Furthermore, B cells can undergo activation through antigen recognition, either through T cell-dependent or T cell-independent mechanisms [32]. 

### 4.3. Resident Immune Cells in the Heart

Resident immune cells within the heart play a crucial role in resident defense against infections and contribute to the repair of locally damaged cardiac tissue caused by sterile inflammation. Among these immune cells, macrophages serve as the principal immune system regulators and are the predominant immune cell population present in steady-state cardiac tissue [33]. Moreover, in stable cardiac tissue, monocytes, dendritic cells, regulatory T cells, and B cells are also present, and they collectively modulate the early immune response [34]. Resident immune cells fulfill a pivotal role not only in safeguarding the body against pathogens but also in orchestrating intricate processes crucial for cardiac development and the preservation of normal tissue functions. Among these immune cells, cardiac macrophages assume multifaceted responsibilities encompassing cardiac electrical conduction, angiogenesis, coronary development, and the maintenance of mitochondrial homeostasis [35]. Essential for sustaining the heart’s homeostasis, cardiac macrophages actively contribute to cellular and extracellular rejuvenation, the clearance of local cellular debris, and the adaptation to tissue strain fluctuations and heightened demands [36]. Absence of resident macrophages accelerates heart failure [PMID: 34645281] Neutrophils are typically absent in healthy cardiac tissue. However, in the context of ischemic or non-ischemic cardiac injury, necrotic cells stimulate both resident immune and non-immune cells residing within the heart, triggering the release of damage-associated molecular patterns (DAMPs) [37]. These DAMPs serve as crucial stimuli for immune cell activation, resulting in the production of pro-inflammatory cytokines and chemokines as well as the recruitment of inflammatory cells from the bloodstream. Following the initial inflammatory phase, neutrophils and macrophages play essential roles in phagocytosing and eliminating dead cells. Moreover, they release cytokines and growth factors, which initiate the reparative process by stimulating the proliferation of damaged myocardial fibroblasts and promoting neovascularization [37].

### 4.4. Recruited Immune Cells Mediate Inflammation Responses in the Heart

The recruitment of immune cells also assumes a significant role in the processes of cardiac injury and repair. Subsequent to cardiac injury, resident cardiac macrophages or inflamed sites secrete inflammatory cytokines and chemokines, such as IL-1, IL-6, and TNF-α. Additionally, cardiac fibroblasts release hematopoietic growth chemokines, including granulocyte colony stimulating factor (GM-CSF), which activate endothelial cells. Subsequently, monocytes and neutrophils are recruited from the circulation to the affected cardiac tissue [38,39]. Recruited monocytes have the ability to differentiate into macrophages, thereby participating in the inflammatory damage response. Furthermore, they release both pro-inflammatory and anti-inflammatory factors to modulate the healing and remodeling processes of the heart. Neutrophils, on the other hand, initiate platelet activation, resulting in thrombosis and coagulation. They initiate the repair process by secreting neutrophil gelatinase-related lipocalin and facilitate endothelial cell activation and cytokine release through the formation of neutrophil extracellular traps [40]. Autoimmune diseases and viral myocarditis have the potential to initiate acute inflammation, which can subsequently advance to subacute and chronic stages. This progressive inflammatory response contributes to the remodeling of myocardial tissue, the development of fibrosis, and the deterioration of myocardial structure and function, culminating in the manifestation of dilated cardiomyopathy. In the context of experimental autoimmune myocarditis, Chihakova [41] et al. demonstrated that the upregulation of macrophage-derived cytokines, including IL-1β, IL-18, IFN-γ, and TNF-β, is associated with acute inflammation and chronic systolic dysfunction observed in dilated cardiomyopathy. Moreover, CD4 T cell-mediated autoimmune responses can induce a Th17-dependent pro-inflammatory cascade, which is linked to the development of dilated cardiomyopathy. T cells secrete IL-17A, which in turn stimulates cardiac fibroblasts to produce GM-CSF, thereby activating monocytes and promoting pro-inflammatory effects [42]. These processes contribute to chronic tissue damage and exacerbate the progression of dilated cardiomyopathy.

## 5. Cellular Communication between Immune Cells and Other Cardiac Cells in DCM

### 5.1. The Endothelium Participates in the Recruitment of Immune Cells

**Figure 2:**
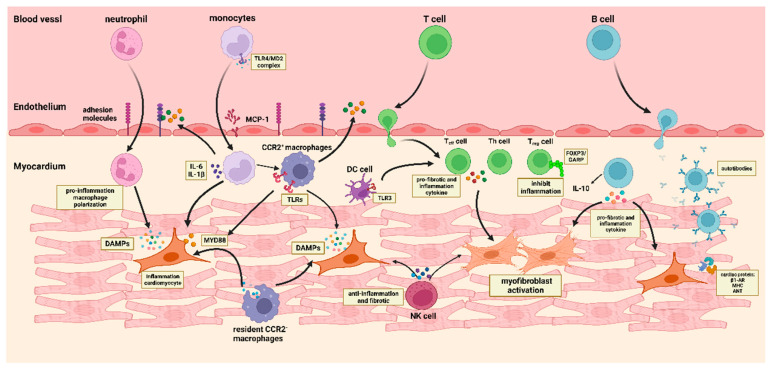
Immune cells interact with cardiac endothelial cells, myocardial cells, and myofibroblasts to play a role in the progression of DCM. Damaged or necrotic cardiomyocytes release DAMPs, which activate signaling pathways, as well as inflammatory factors and DAMPs that stimulate endothelial cells to secrete various adhesion molecules and cytokines, thus facilitating the migration of immune cells. Resident cardiac macrophages proliferate and secrete cytokines or chemokines to recruit monocytes and neutrophils from the peripheral blood. Monocytes recruited into the myocardium release cytokines to participate in the repair and remodeling of cardiac injury, as well as transform into macrophages to participate in the inflammatory injury response of the myocardium. Migration of various anti- and pro-inflammatory T cells to sites of myocardial injury in response to antigen presentation by dendritic cells. Cytokines released by Treg cells and Th cells intensify the inflammatory response and activate myofibroblasts, while Treg cells, through the expression of FOXP3/GARP, are able to reduce the severity of the immune inflammatory response. B cells contribute to the immune regulation of DCM by secreting chemokines, cytokines, and autoantibodies. Myofibroblasts are activated by inflammatory mediators and fiber growth factors released by various immune cells to promote myocardial fibrosis. Natural killer cells act to reduce inflammation and protect the heart.

During heart failure, endothelial cells in microvessels display an augmented expression of molecules including P-selectin, E-selectin, intracellular cell adhesion molecule-1 (ICAM-1), and vascular cell adhesion molecule-1 (VCAM-1), which facilitate the migration of immune cells, for instance B cells, T cells, natural killer cells, and monocytes, to cardiac myocytes [43,44]. Endothelial cells are commonly activated by pro-inflammatory cytokines, such as TNF-α and IL-6, which are secreted by immune cells upon encountering pathogens [45]. Additionally, endothelial cell activation can also be induced by the release of DAMPs from stressed and dying cells. The binding of DAMPs to endothelial cells triggers the upregulation of pro-inflammatory signaling pathways, including NF-κB, MAPK, and interferon regulatory factor 3 (IRF3) signaling. Consequently, this leads to increased production of adhesion molecules such as E-selectin, ICAM-1, and VCAM-1, as well as cytokines like IL-6, IL-8, and IFNγ [46] (Figure 2).

### 5.2. The Interplay between Immune Cells and Cardiomyocytes in DCM

Cardiomyocytes are a special type of cell located in the heart, exhibiting specific characteristics that make them distinct. Cardiomyocytes are large, with a great deal of mitochondria and sarcomere occupying the majority of the cell volume, and are essential for preserving cardiac function, involving themselves in the immune response to viral infection, and in the repair of cardiac injuries [47]. During the initial stages of inflammation, cardiomyocytes suffer necrosis, releasing DAMPs, which can activate the inflammatory signaling pathway when interacting with Toll-like receptors, triggering the production of various chemokines, such as C-C motif chemokine ligands (CCL-2, CCL-5, and CCL-7), and C-X-C chemokine ligands (CXCL-1, CCL-2, and CCL-8) [48]. Following the increased chemokines, resident CCR2^+^ macrophages are activated, thus prompting the release of pro-inflammatory cytokines like TNF-α and IL-1. These cytokines promote endothelial cell proliferation and draw activated CCR2^+^ monocytes and neutrophils to the site of inflammation. Additionally, CCR2^−^ macrophages secrete anti-inflammatory cytokines, such as IL-10 and TGF-β, which induce the transformation of fibroblasts into myofibroblasts (Figure 2). This phenotypic transition leads to the accumulation of extracellular matrix components and collagen within the myocardium, ultimately resulting in the development of cardiac fibrosis [49,50].

### 5.3. Immune Cells Contribute to Fibrosis in DCM by Activating Myofibroblasts

Myocardial fibrosis, characterized by the activation of myofibroblasts and the subsequent accumulation of extracellular matrix proteins, is a prevalent feature in various heart diseases. Immune cells, such as macrophages, mast cells, and lymphocytes, can be recruited and activated in the remodeled heart, which may significantly influence the stimulation of myofibroblasts. These immune cells secrete a variety of fibrosis mediators, such as cytokines, growth factors, and matricellular proteins, which play a role in the development and progression of myocardial fibrosis [51,52]. Monocytes and macrophages can transform into myofibroblasts in response to various cytokines, resulting in the secretion of inflammatory mediators and fibrogenic growth factor [53] (Figure 2). 

In addition, activated lymphocytes, including CD8^+^, Th1, Th2, Th7, and T-reg CD4^+^ subpopulations, have been shown to contribute to fibrogenesis, remodeling, and dysfunction of the heart. Particularly, Th1 cells can activate cardiac fibroblasts by stimulating TGF-β [54,55]. A study conducted in mice demonstrated that TGF-β can trigger kinase-1-mediated rapid Wnt protein secretion, thereby inducing TGF-β-mediated myofibroblast differentiation and cardiac fibrosis. These processes promote cardiac remodeling in autoimmune myocarditis, ultimately leading to dilated cardiomyopathy [56]. In patients with DCM, increased mRNA levels of myocardial IL-6 and TNF-α have been associated with collagen accumulation and gelatinase expression. Elevated TNF-α expression in mice has been linked to the expansion of cardiac mast cells, which can contribute to fibrogenic actions in the myocardium [57,58].

## 6. Regulatory Mechanisms of Immune Cells in DCM

### 6.1. Monocytes and Macrophages in DCM

In a physiological state, the heart harbors a minimal number of monocytes. In humans, three distinct monocyte subpopulations have been identified: classical (CD14^++^CD16^−^), intermediate (CD14^++^CD16^+^), and non-classical (CD14^+^CD16^+^) monocytes. Conversely, mouse monocytes can be categorized into two subgroups based on the expression of Ly6C: classical Ly6Chigh monocytes and non-classical Ly6Clow monocytes [59,60]. When the heart is harmed, classical monocytes are usually drawn to the inflammatory region, and non-classical monocytes stay in endothelial cells to maintain homeostasis [59]. Monocytes activate the TLR4/MD-2 complex, which leads to the release of inflammatory cytokines. MD-2 is a 20–25 kDa protein that can be found in plasma or associated with the membrane, and it is in close proximity to TLR4 [61,62]. Feldtmann et al. [63] demonstrated that MD-2 can induce a pro-inflammatory state in monocytes and endothelial cells through TLR4/NF-κB signaling in patients with DCM. MD-2 activation of endothelial cells results in the secretion of monocyte-chemoattractant protein-1 (MCP-1) and increased expression of adhesion molecules CD54, CD106, and CD62E, thereby promoting enhanced monocyte recruitment and the progression of DCM. When MD-2 activates endothelial cells, it results in the release of MCP-1 and an increased presence of adhesion molecules CD54, CD106, and CD62E, thus facilitating the recruitment of monocytes and the advancement of DCM. MCP-1 has been identified as a crucial chemokine in monocyte mobilization and recruitment to inflammatory sites. It is also known to induce the expression of adhesion molecules on monocytes and the secretion of interleukin 6 (IL-6) and interleukin 1β (IL-1β) [64,65]. Elevated levels of IL-6 and IL-1β in individuals with dilated cardiomyopathy (DCM) can lead to cardiomyocyte apoptosis and impaired systolic function of the heart [66,67]. Lehmann et al. [68] found that DCM patients with a more serious decline in left ventricular function had increased levels of MCP-1 messenger RNA than those with less severe symptoms, implying that the raised MCP-1 concentrations could be responsible for myocyte damage through monocyte activation and attraction. Furthermore, Kobayashi et al. [69] found a positive relationship between the expression of myocardial MCP-1 and the severity of cardiac dysfunction in individuals with DCM, with higher MCP-1 expression associated with worse cardiac function.

Most cardiac macrophages derive from blood monocytes and the yolk sac (YS), and they typically appear as spindle cells scattered within cardiomyocytes, fibroblasts, and endothelial cells [70,71,72]. The expression of CCR2 allows for the classification of cardiac macrophages into two subsets: CCR2^+^ macrophages, deriving from embryonic hematopoietic lineages, and CCR2^−^ macrophages, originating from adult hematopoietic lineages. The CCR2^+^ macrophages, originating from monocytes, are mainly responsible for cardiac fibrosis and the inflammation response [72,73]. Bajpai et al. [74] demonstrated that tissue-resident cardiac CCR2^+^ macrophages play a crucial role in initiating the inflammatory response to myocardial injury, which is activated through the myeloid differentiation primary response 88 (MYD88) signaling pathway. Upon activation, these tissue-resident CCR2^+^ macrophages secrete inflammatory chemokines and cytokines that attract monocytes and neutrophils to the site of myocardial inflammation. The MYD88 signaling pathway is widely recognized as a major mediator of the effects of DAMPs released from dying cells, including several signaling pathways involving Toll-like receptors (TLRs; TLR2, TLR4, and TLR9) and the IL-1 receptor. However, the specific receptor responsible for activating tissue-resident CCR2^+^ macrophages in the context of myocardial injury remains uncertain [75]. In addition, tissue-resident CCR2^+^ macrophages are responsible for regulating the infiltration of neutrophils and monocytes into the myocardium. This is achieved by controlling the transendothelial migration of neutrophils and the recruitment of monocytes from the periphery to the heart [74]. In contrast, CCR2^−^ macrophages, predominantly located in viable myocardium, have the capacity to regulate tissue repair. Wong et al. [76] demonstrated that the absence of CCR2^−^ macrophages in mice with DCM led to a rapid mortality rate, obstructed ventricular remodeling, and reduced coronary angiogenesis. Through focal adhesion complexes, macrophages and cardiomyocytes interacted, and the myocardial stretch stimulated the macrophages via a TRPV4-dependent pathway, controlling the expression of growth factors. Furthermore, cardiac macrophage function is largely determined by the presence of M1 and M2 macrophages. M1 macrophages are recognized for their secretion of pro-inflammatory cytokines, including TNF-α, IL-1β, and IL-6, which play a role in the acute inflammatory response. In contrast, M2 macrophages are characterized by their production of anti-inflammatory cytokines, such as vascular endothelial growth factor (VEGF) and TGF-β, which are crucial for cardiac fibroblast activation, angiogenesis, and wound healing [77,78,79]. Nakayama et al. [80] revealed that the presence of anti-inflammatory M2 macrophages, identified by CD163^−^-positive infiltrates, was an independent predictor of cardiac fibrosis and was associated with a poorer long-term prognosis for patients with DCM. Moreover, a greater number of infiltrating CD3^−^-, CD68^−^-, and CD163^−^-positive cells was associated with a poorer outcome. 

Previous studies have provided evidence that upon stimulation, cardiomyocytes secrete inflammatory cytokines and chemokines, as well as pathogen-associated molecular patterns (PAMPs) and DAMPs. The recognition of PAMPs/DAMPs by pattern recognition receptors (PRRs) leads to the activation and expansion of resident immune cells, along with the recruitment of circulating immune cells from the bone marrow, thereby amplifying the inflammatory response [81]. Cardiac tissue has a large population of resident macrophages, which can be activated when they detect PAMPs/DAMPs through a variety of pattern recognition receptors (PRRs), which are further increased during injury by the infiltration of circulating monocytes [49]. Activated monocytes release cytokines (such as IL-6 and IL-1β) into the peripheral circulation to regulate the myocardial inflammatory response and, under the influence of the chemokine MCP-1, migrate to the site of myocardial inflammation [68,72,82,83]. Additionally, monocyte-derived CCR2^+^ macrophages sustain the inflammatory environment within the myocardium by recruiting and supporting the growth of monocytes, while CCR2^−^ macrophages contribute to the repair of damaged myocardium by replenishing the local tissue [84]. When the pro-inflammatory and anti-inflammatory phases are not balanced, it can have a detrimental effect, including reduced systolic activity and unfavorable alterations in the left ventricle [85]. Furthermore, the polarization of macrophages to the M2 phenotype may be associated with ventricular remodeling in DCM patients [80]. A recent study employing single-cell RNA sequencing (scRNA-seq) analysis of CD45^+^ cells isolated from mouse hearts at different stages of experimental autoimmune myocarditis (EAM) has shed light on the transition from myocarditis to cardiomyopathy. The study revealed that macrophages during the inflammatory stage may play a role in antigen processing and presentation, as well as in the response to IFN-γ, whereas macrophages during the myopathy phase may be associated with the mitogen-activated protein kinase (MAPK) cascade, TNF signaling, and NF-κB signaling [24]. Additionally, plasminogen activator inhibitor-1 (PAI-1) exerts a cardioprotective effect by inhibiting cardiac fibrosis through the plasminogen-mediated and TGF-β pathways. In a retrospective study, Baumeier et al. [86] investigated the expression of plasminogen activator inhibitor-1 (PAI-1) in endomyocardial biopsies (EMBs) from patients with dilated cardiomyopathy (DCM). Their findings suggested that PAI-1 not only inhibits fibrosis by blocking TGF-β signaling and myofibroblast activation but also promotes a transition from pro-inflammatory M1 macrophages to anti-inflammatory M2 macrophages. In an animal study, ICAM-1, a cell adhesion molecule crucial for leukocyte recruitment, was found to facilitate macrophage adherence to cardiomyocytes. This adherence can lead to reduced cardiomyocyte contractility through the expression of TGF-β, oxygen free radicals, and nitric oxide [87,88]. Gasparini et al. [89] conducted a study to investigate the pathological processes of canine DCM and proposed that macrophages were involved in the cardiac remodeling processes. This is attributed to the expression of ICAM-1, TGF-β, and vascular endothelial growth factor, which may be responsible for cardiac dilation and dysfunction.

### 6.2. T Cells in DCM

T cells, the primary cells in cellular immunity, are responsible for producing cytokines in order to mediate immune reactions [90]. Based on the cytokines released, T cells can be classified into T helper 1 (Th1), Th2, Th17, T follicular helper (Tfh), and regulatory T cells. These types of T cells have distinctive developmental and regulatory pathways, and they play varied roles in immunity and diseases related to the immune system [91]. Studies have demonstrated that different T cells are involved in myocardial inflammation and cardiac damage and have an effect on the progression of DCM [92,93]. Furthermore, research has revealed that T lymphocytes contribute to the development of DCM through direct cytotoxic effects, activation of other immune cells, and support of pathogenic antibody production by B cells [94] Particularly, CD4+ T cells have been identified as a major contributor to cardiac fibrosis, primarily through pro-inflammatory Th1 and Th17 subsets and their associated cytokines, such as IL-6, IL-1β, TNF, and IL-17, which can activate resident cardiac fibroblasts and promote fibrosis [95,96,97]. Additionally, elevated levels of peripheral Th1, Th7, and Th22 cells, as well as their corresponding cytokines IFN-γ, IL-17, and IL-22, have been observed in patients with DCM, indicating their potential involvement in the disease [98,99]. Veronika Nindl and her colleagues have revealed that IFN-γ is a key effector cytokine that triggers the inflammatory process and that the combination of IFN-γ and IL-17A is necessary for the transition from autoimmune myocarditis to DCM [100]. IFN-γ has a pro-inflammatory role, which includes antiviral activity and the activation of Th1 cells and cytotoxic T cells [101]. However, previous studies indicated that IFN-γ can help reduce acute inflammation and that mice deficient in IFN-γ have been observed to have an increased inflammatory response when exposed to a cardiac myosin (CM)-induced model of EAM [102,103,104]. Afanasyeva et al. [105] discovered that the absence of IFN-γ in murine myocarditis facilitates the proliferation of CD4^+^CD44^+^CD25^+^ T cells, which in turn intensifies cardiac inflammation and causes cardiac dysfunction and a higher likelihood of developing dilated cardiomyopathy than in wild-type mice. 

Research conducted by Guo and colleagues has highlighted the significant role of CD4+ effector T cells producing IL-22, also known as Th22 cells, in the progression of chronic myocarditis and dilated cardiomyopathy in mice infected by CVB3 [106]. IL-22 has been found to be a cardioprotective cytokine with the capacity to inhibit myocardial fibrosis and has been associated with the expression of biomarkers such as COL1-A1, COL3-A1, and MMP9 [106]. In contrast, Th17 cells are a separate subset of CD4+ T cells, distinct from Th1 and Th2 cells, and generate IFN-γ and IL-4. However, the precise involvement of Th17 responses in the progression of human dilated cardiomyopathy is still not fully understood. Th17 cells are characterized by the secretion of IL-17, a proinflammatory cytokine [107]. Studies have revealed that mice lacking IL-17A are protected from cardiomyopathy and exhibit a reduction in the number of neutrophils and monocytes in the myocardium [108]. Furthermore, IL-17A induces a proinflammatory state in monocytes by stimulating GM-CSF production from cardiac fibroblasts [109]. In human myocarditis, Myers et al. discovered a Th17 cell immunophenotype linked to human myocarditis/DCM, which was characterized by increased CD4^+^IL-17^+^T cells and Th17-promoting cytokines, such as IL-6, TGF-β, and IL-23, along with GM-CSF-secreting CD4^+^T cells. It was suggested that this Th17 phenotype was connected to the effects of cardiac myosin on CD14^+^ monocytes, TLR2, and heart failure [110]. IL-17 exerts diverse effects, including the induction of proinflammatory cytokines (such as IL-6 and TNF-α), chemokines such as macrophage chemoattractant protein-1 (MCP-1) and macrophage inflammatory protein-2 (MIP-2), as well as matrix metalloproteases. These molecular mediators facilitate tissue infiltration and the subsequent destructive processes [111]. It has been suggested that IL-17 is a causative factor in DCM as it induces the migration of primary cardiac fibroblasts in a MMP-1-dependent manner. This suggests that IL-17 may be involved in the remodeling of the myocardium, which could be a factor in the development of DCM [112,113].

Tregs, a subset of CD4 T cells, play a major role in peripheral tolerance, avoiding autoimmune diseases, and moderating chronic inflammatory diseases [114]. These cells are characterized by the expression of biomarkers such as forkhead box P3 (FOXP3), glycoprotein a repetitions predominant (GARP), and CD25, in addition to CD4, and are responsible for maintaining immune balance by inhibiting the development of effector T cells [115,116,117]. Tang et al. [118] observed a notable reduction in the levels of FOXP3 mRNA and protein expression in peripheral blood mononuclear cells from individuals with dilated cardiomyopathy (DCM) when compared to healthy subjects. This decrease in FOXP3 expression may lead to a decline in the population of regulatory T cells (Tregs), potentially contributing to the progression of DCM. In another study, Wei et al. [98] indicated that the levels of Th1, Th17, and their related cytokines (IFN-γ and IL-17) were higher in patients with DCM. At the same time, the number of Tregs, TGF-β1 levels, and the expression of FOXP3 and GARP were significantly decreased in DCM patients. Moreover, the suppressive capacity of CD4^+^CD25^+^GARP^+^Tregs was impaired in DCM patients, resulting in decreased secretion of TGF-β1 and increased secretion of IFN-γ and IL-17. TGF-β1 is a cytokine with multiple functions, which can be seen in its ability to modulate FOXP3 expression and influence the activity of Tregs through SMAD2/3 phosphorylation, thus having a beneficial effect in the initial stages of inflammation [119]. A novel T cell subset, CD4^+^LAP^+^Tregs, has recently been identified and has been shown to possess the ability to suppress the immune response [120]. Zhu et al. [121] demonstrated that the number of CD4^+^LAP^+^Tregs in patients with DCM is lower than normal and their inhibitory activity is impaired. Furthermore, it was found that CD4^+^LAP^+^Tregs can inhibit the proliferation of Tresps without affecting the secretion of TNF-a and IFN-γ. Additionally, CD4^+^LAP^+^ Tregs can directly suppress B cell proliferation and IgG production through contact with B cells, implying that soluble and contact-dependent mechanisms are involved in the suppression of B cell proliferation by CD4^+^LAP^+^ T cells. CD4^+^LAP^+^ Tregs have the capacity to inhibit B cell proliferation and IgG production through both soluble and contact-dependent mechanisms. In addition, alterations in the number of follicular regulatory T cells (Tfr) that circulate in the body could potentially have a significant effect on the immunological modulation of DCM. Liu et al. [122] discovered that a decrease in Tfr cells may be connected to the disturbance of the immune balance in individuals with DCM, prompting the activation of T cells and germinal center B cells. This could result in the secretion of a large amount of cytokines and immunoglobulins, ultimately causing a worsening of cardiac performance and myocardial damage.

Research has provided evidence of the substantial involvement of T cells in the transition from autoimmune myocarditis to dilated cardiomyopathy, primarily through the Th1- and Th17-driven pathways [100,123]. Studies have consistently shown significantly elevated levels of Th1, Th17, and Th22 cells in the peripheral blood of individuals diagnosed with dilated cardiomyopathy [99,124]. Furthermore, Studies have demonstrated that the activation of Th22 cells in dilated cardiomyopathy patients is linked to a heightened expression of the aryl hydrocarbon receptor (AHR) in the peripheral blood, potentially playing a role in the pathogenesis of DCM [99]. CD4^+^ regulatory T cells (Tregs) have been found to reduce the intensity of acute cardiac inflammation and restrain the development of myocarditis into dilated cardiomyopathy. These specialized cells have the ability to reduce inflammation by producing anti-inflammatory cytokines, inhibiting activated lymphocytes, and regulating pro-inflammatory cytokines such as IL-2 [125]. Treg cells are characterized by the presence of the transcription factor FOXP3, and their immunosuppressive function can be assessed by the expression of GARP. GARP is activated by FOXP3 and possesses the capability to restrain immune responses. Perturbations in GARP signaling can result in enhanced immune responses [115,126]. Evidence has revealed that CD4+CD25+GARP+ Tregs derived from humans can reduce the proliferation of Tregs and influence the secretion of pro-inflammatory cytokines, such as IFN-γ and IL-17, in people suffering from dilated cardiomyopathy [98]. In addition, autoimmune and inflammatory processes have been suggested to contribute to the emergence of DCM. Research conducted on the experimental autoimmune myocarditis model revealed that a lack of IFN-γ was associated with an exacerbated development of myocarditis in DCM, which was correlated with a failure to mature regulatory T cells [99]. Lindberg et al. [127] report that patients with DCM have a significantly reduced activation of IFN-γ^+^CD4^+^T cells and a decreased production of IL-10. These two cytokines, IFN-γ and IL-10, are essential for the body’s capacity to mount protective immune responses and to preserve self-tolerance. IFN-γ and IL-10 are both indispensable for the body’s ability to generate defensive immune responses and to maintain self-tolerance. Furthermore, miRNAs play a crucial role in the progression of T-lymphocyte activation, proliferation, development, and aging. Zeng et al. [128] revealed that the reduction of miRNA-451a is associated with the activation and proliferation of CD4+ T cells in DCM patients, which is due to the inhibition of the transcription factor Myc, thus suggesting that miRNA-451a plays a role in the immunopathogenesis of DCM. Recent animal studies have revealed that Toll-like receptor 3 (TLR3) can impede the progression from myocarditis to inflammatory DCM in coxsackievirus B3-infected mice by suppressing acute viral replication and reducing IL-4 levels in the heart. The lack of TLR3 caused a heightened production of cytokines related to a Th2 response, such as IL-4, IL-10, IL-13, and TGF-β1, consequently instigating an immunoregulatory environment in the cardiac tissue [129].

### 6.3. B Cells in DCM

B cells are critical for the maintenance of the immune system, and any abnormalities in their numbers or function can lead to autoimmune problems in individuals with DCM [130,131,132]. In a viral myocarditis mouse model, Li et al. [133] showed that B cells exacerbate myocardial inflammation by inhibiting the anti-inflammatory M2 macrophage phenotype. Guo et al. [134] found a higher frequency of IL-10-producing Bregs in the peripheral blood of DCM patients, suggesting their significant role in the etiology of DCM. Jiao et al. [135] also observed a reduced population of CD24hiCD27+ B cells, which produce IL-10, in DCM patients compared to healthy controls. This reduction was associated with a diminished ability to suppress TNF-α production by CD4+CD25- Tconv cells and impair Treg differentiation. Thus, their findings suggest a protective role for Bregs in DCM development, which is closely correlated with left ventricular (LV) function. Importantly, B cells play a vital role in the etiology of DCM as they are capable of synthesizing antibodies against cardiac proteins, including β1 adrenergic receptor (β1-AR), myosin heavy chain (MHC), adenine nucleotide translocator (ANT), and others [136,137,138]. Tang et al. [139] demonstrated that a decrease in the proportion of B1 cells is strongly correlated with a reduction in left ventricular ejection fraction (LVEF), elevated NT-proBNP levels, and increased levels of β1-AR autoantibodies, indicating that a decline in B1 cell numbers can exacerbate cardiac dysfunction and the development of heart failure in individuals with DCM. The production of autoantibodies that are directed against cardiac antigens is a consequence of the breakdown of immune tolerance to cardiac autoantigens and is a significant indication of an immune system disorder in patients with dilated cardiomyopathy. Autoantibodies targeting β1-AR not only increase the risk of heart failure but also have a deleterious effect on myocardial remodeling due to their sympathetic-like effects when binding to β1-AR [137]. In addition, β1-AR autoantibodies can lead to cardiomyocyte apoptosis, which is associated with a rise in fatal ventricular arrhythmias, sudden death, and all-cause and cardiac mortality in patients with DCM [140,141,142].

B cells are a major contributor to cytokines, including pro-inflammatory agents like TNF-α, lymphotoxin, IL-6, and IL-17, and anti-inflammatory molecules like IL-10 [143]. Yu et al. [144] discovered that the amount of TNF-α-secreting B cells was significantly higher in DCM patients, and these B cells were associated with myocardial fibrosis. Additionally, TNF-α-secreting B cells were positively correlated with serum procollagen type III (PCIII), suggesting that these B cells can promote PCIII accumulation in cardiac fibroblasts through the secretion of TNF-α. Siwik et al. [145] demonstrated that TNF-α is capable of promoting cardiac fibroblast proliferation and collagen synthesis via ERK1/2 signaling in vitro. For DCM patients with previous acute viral myocarditis, Yuan et al. [138] discovered that Th17 cells can activate B cells, resulting in the production of antiheart antibodies due to the release of IL-17. IL-17 binds to the IL-17 receptor on B cells, initiating the B cell-activating factor signaling pathway, which contributes to the transition from acute viral myocarditis to DCM. However, during the progression from acute viral myocarditis to DCM, the production of IL-17 is inhibited by INF-γ, IL-4, and T-bet. Concurrently, the activation of Toll-like receptor 3 (TLR3) through dsRNA viral intermediates stimulates the production of IL-4. B cells are vital as they not only generate a wide range of cytokines such as TNF-α, lymphotoxin, IL-6, and IL-17, which are proinflammatory mediators, but also manufacture anti-inflammatory mediators like IL-10. The interplay of these cytokines has both beneficial and detrimental effects on myocardial fibrosis [108,143,146]. Notably, DCM patients exhibit an increased presence of TNF-α-secreting B cells, suggesting their involvement in myocardial fibrosis. Moreover, It has been observed that the number of TNF-α-secreting B cells is inversely proportional to the left ventricular ejection fraction (LVEF) of patients suffering from DCM and positively associated with left ventricular end-diastolic dimension, NT-proBNP, and procollagen type III [144]. The Bregs are known to be able to regulate the immune system by producing regulatory cytokines and having direct contact with T cells [147]. These regulatory B cells are typically characterized by the presence of IL-10. However, in the context of dilated cardiomyopathy (DCM), a reduction in the frequency of interleukin-10-producing regulatory B cells (Bregs) (CD24hiCD27+) is observed, and these cells exhibit an impaired ability to suppress the production of TNF-α by T effector cells [135,148].

### 6.4. Natural Killer Cells in DCM

NK cells, a part of the innate lymphoid cell (ILC) population, have been observed to be abnormal in terms of their number and function in conditions such as myocarditis, inflammatory dilated cardiomyopathy, and heart transplant rejection [149]. NK cells have a significant function in protecting against acute viral pathogens related to myocarditis. Research conducted by Yokoyama and Kanda [150,151] has shown a reduced activity of NK cells in patients with dilated cardiomyopathy, suggesting a close relationship between NK cell suppression and the pathophysiology of chronic myocarditis, which may be associated with the development of mild dilated cardiomyopathy. Ong et al. [152] conducted an animal study and demonstrated that NK cells may act as a protective factor against cardiac fibrosis in mice with EAM. They have the effect of stifling collagen production in cardiac fibroblasts and holding back the assembly of inflammatory cells in the heart. Furthermore, NK cells are capable of regulating eosinophil numbers by inducing eosinophil apoptosis; however, if NK cells are depleted, the condition deteriorates and cardiac eosinophil infiltration occurs. In addition, NK cells are capable of preventing viral replication by detecting and destroying the resident cells that are infected, and they are responsible for the initial production of type I interferons, such as IFN-α, IFN-β, and IFN-γ, which initiated the anti-viral inflammatory cascade [153,154]. Moreover, activated NK cells release IFN-γ, which can suppress the release of Th2 cytokines from ILC2 populations. ILC2 cells induce the secretion of eotaxins from cardiac fibroblasts, and NK-derived IFN-γ can indirectly reduce local eotaxin concentrations, thereby reducing the ability of eosinophils to migrate to the heart. This ultimately helps mitigate the detrimental impact of eosinophils on the myocardium [155]. In conclusion, NK cells have a significant role in the regulation of disease and dysregulation, and their biology has a profound impact on clinical outcomes. Further research is required to gain a comprehensive understanding of the role and mechanisms of NK cells in patients with DCM. Further investigation is necessary to acquire an extensive comprehension of the role and mechanisms of NK cells in individuals with DCM. 

### 6.5. Neutrophils in DCM

As a significant element of the innate immune system, neutrophils have been shown to be involved in cardiac remodeling caused by various external or internal triggers in patients with myocardial infarction 29,167,168]. However, the role of neutrophils in dilated cardiomyopathy (DCM) remains insufficiently investigated, and the immune response processes involved in DCM remain largely unknown. Neutrophils, as recruited immune cells, typically migrate from the circulation to sites of cardiac inflammation, contributing to the inflammatory response by releasing cytokines and chemokines [38]. Neutrophil gelatinase-associated lipocalin (NGAL), initially discovered in neutrophils, has been identified in various immune cells [156,157]. Tawfeek et al. [158] conducted a study to measure the levels of NGAL in the plasma of children with heart failure caused by idiopathic dilated cardiomyopathy (IDCM). They observed a significant elevation of plasma NGAL levels in children with IDCM-associated heart failure, which was not associated with myocardial function indices. Moreover, studies have indicated a correlation between the neutrophil-to-lymphocyte ratio (NLR) and the severity of chronic heart failure in patients with idiopathic dilated cardiomyopathy [159]. Araújo et al. [160] discovered that NLR values equal to or above 5.2, as well as lymphocyte levels below 1000 μL, were associated with a negative prognosis and increased likelihood of mortality or the requirement for cardiac transplantation in pediatric and adolescent dilated cardiomyopathy. Further investigations are needed to gain a better understanding of the involvement of neutrophils in DCM and their potential as prognostic markers.

### 6.6. Dendritic Cells in DCM

Dendritic cells (DCs), which account for roughly 1% of the total leukocyte population in the heart, act as a bridge between the innate and adaptive immune systems [161]. In a similar way to cardiac resident macrophages, DCs become activated when they encounter foreign or pathogenic antigens or signals of tissue inflammation. They subsequently transport cellular debris and antigens to T cells, thereby enabling T cells to recognize and respond to pathogens by undergoing activation [30,72]. Although the precise involvement of dendritic cells in heart failure remains incompletely understood, accumulating evidence suggests their likely contribution to myocarditis and DCM. Pistulli et al. [162] reported a decreased number of myocardial DCs in heart biopsies obtained from symptomatic DCM patients, which was associated with a poor short-term prognosis in terms of left ventricular ejection fraction. This reduction in chronic heart failure might be due to damage to myocardial tissue, cell death, and inadequate vascularization. Under normal conditions, DCs are recognized for inducing peripheral tolerance by rendering autoreactive T cells anergic or promoting the generation of regulatory T cells from naive T cells [163]. However, under conditions of tissue inflammation or genetically altered activation thresholds of DCs, the activation of self-reactive T cells by DCs can lead to the development of autoimmunity [164,165]. It has been proposed that T cells activated by DCs and the associated inflammatory processes can result in augmented myocardial damage due to fibrosis and remodeling [166]. Athanassopoulos et al. [167] suggested that the total number of DCs in the blood is increased in patients with end-stage heart failure, particularly in those with dilated cardiomyopathy. Investigating the characteristics of DCs in peripheral blood may provide novel insights into the pathogenesis of idiopathic dilated cardiomyopathy in humans.

## 7. The Role of Immune Cells in Other Cardiomyopathies

In arrhythmogenic cardiomyopathy (ACM), infiltration of lymphocytes and macrophages into the myocardium can lead to inflammatory reactions, which may result in immune-mediated arrhythmias or damage to the myocardial cells [168]. In patients diagnosed with hypertrophic cardiomyopathy (HCM), there was a notable reduction in the presence of macrophages, monocytes, dendritic cells (DC), Th1 cells, Treg cells, and plasma cells, whereas CD8^+^ T cells, basophils, fibroblasts, and platelets exhibited significantly heightened levels. The STAT3-related pathway, alongside CD163^+^LYVE1^+^ macrophages, emerged as pivotal factors and immune cells implicated in the regulation of the immune mechanism underlying HCM [169,170]. Restrictive cardiomyopathy (RCM) is a damaging myocardial disorder characterized by impaired diastolic functioning due to flawed muscle relaxation and hardened myocardium, leading to restricted ventricular filling. It is estimated that around 30% of restrictive cardiomyopathy cases are familial and linked to genes such as cTnT, cTnI, MyBP-C, MYH7, MYL2, MYL3, DES, LNMA, and FLNC [171]. Left ventricular non-compaction cardiomyopathy (LVNC) is a rare cardiac disorder arising from an aberration in the normal compaction process of the central muscle wall within the left ventricle during fetal development, resulting in excessive trabecular formation. The regulation of immune cells in LVNC remains insufficiently investigated, notwithstanding the detection of numerous gene mutations in LVNC that bear similarities to those observed in other cardiomyopathies, notably dilated cardiomyopathy [172].

## 8. Immunotherapy for DCM

In accordance with guidelines, the pharmacological management of dilated cardiomyopathy (DCM) is comparable to that of heart failure. Nevertheless, there is currently no evidence-based specific treatment for DCM, and in certain instances, HTx represents the sole alternative when medical interventions prove inadequate in halting the progression of heart failure [173]. Consequently, exploring novel therapeutic strategies that target immune system dysregulation may offer promising therapeutic options or adjunctive therapies for patients suffering from heart failure associated with DCM based on the cellular function of various cell types (Table 1).

### 8.1. Immunosuppression Therapy

Immunosuppressive therapy involves the utilization of immunosuppressants to impede the growth and activity of cells related to the immune response (such as T cells, B cells, and macrophages), thereby decreasing the antibody immune response [176]. Prednisolone and azathioprine are among the most commonly prescribed immunosuppressants. In adults, although two early clinically randomized controlled trials demonstrated that immunosuppressive therapy does not significantly enhance cardiac function in patients with myocarditis, the efficacy of this approach remains contentious. It should be noted that immunosuppressive therapy may not confer benefits to all adult patients with myocarditis [177,178,179]. As reported by Frustaci et al. [180], individuals with a viral presence in the myocardium are typically unresponsive to treatment with prednisolone and azathioprine, whereas those exhibiting cardiac autoantibodies in their system and no viral genome in the myocardium are more likely to respond positively to immunosuppression. Furthermore, findings from a randomized study indicated that a 6-month regimen of prednisolone and azathioprine can significantly improve left-ventricular ejection fraction and result in a substantial reduction in left-ventricular dimensions and volumes in individuals with virus-negative inflammatory cardiomyopathy [181]. In children, various immunosuppressants have been investigated for the treatment of DCM and myocarditis, with prednisolone being the most commonly used agent [182]. Kleinert et al. [183] showed that cyclosporine and prednisolone could enhance left ventricular performance in children suffering from DCM. Furthermore, recent research has revealed that immunosuppression therapy (azatioprine and prednisone) significantly raised the left ventricular ejection fraction and cardiac output, even in the presence of viral genomes, in children with myocarditis [184]. However, a meta-analysis concluded that immunosuppressive therapy does not have a considerable effect on the prognosis of children with acute myocarditis, and thus, there is not enough evidence to recommend its regular use [185]. Therefore, a prospective, large-sample, multicenter randomized controlled trial is necessary to further clarify the therapeutic role of immunosuppression in DCM.

### 8.2. Intravenous Immunoglobulin

Intravenous immunoglobulin (IVIg) has the potential to bring about a range of effects on the host immune system, such as promoting the production of anti-inflammatory cytokines, setting off anti-idiotypic activities, elevating Fcgamma receptor saturation, and expressing the inhibitory FCgRIIB. Additionally, IVIg can interact with microbial particles, promote sequestration of self-antigens, and interfere with B and T cell regulation [186,187]. Dennert et al. [188] demonstrated a significant reduction in viral load and improvement in cardiac function following the administration of intravenous immunoglobulin (IVIg) at a dosage of 2 g/kg in patients with DCM associated with a high parvovirus B19 (PVB19) viral load in the heart. Heidendael et al. [189] suggested that, compared to untreated children, those who received IVIg did not show an increased transplant-free survival rate within a 5-year period but exhibited improved systolic left ventricular function and a higher rate of recovery during the same duration. Additionally, Prasad et al. [190] discovered that those who underwent IVIg therapy had an augmented left ventricular ejection fraction and a diminished left ventricular end-diastolic diameter six months following treatment in comparison to those who did not receive IVIg. However, despite the potential of IVIg therapy, not all studies have reported a favorable impact on hemodynamic or cardiac conditions. McNamara et al. [191] conducted a large-scale randomized trial to study the effects of IVIg therapy and found no significant improvement in left ventricular ejection fraction among patients with new-onset dilated cardiomyopathy, even after 6 and 12 months of treatment. The short-term prognosis of IVIg therapy in this context remains uncertain. An experimental study of IVIg as a supplement to standard therapy in individuals suffering from idiopathic chronic dilated cardiomyopathy and persistent B19V infection failed to demonstrate any further benefit on cardiac systolic function or functional capacity after 6 months of treatment in comparison to conventional medical therapy, which was evaluated in a randomized, double-blind, placebo-controlled, and single-center trial [192]. 

### 8.3. Immunoadsorption

Immunoadsorption (IA), a process of removing circulating antibodies, has been demonstrated to be an effective treatment for a variety of autoimmune diseases, such as autoimmune encephalitis, connective tissue disease, and rheumatic diseases [193,194,195]. Therefore, IA may be a promising therapeutic option for DCM, as it can help regulate the humoral immune system and intervene in the autoimmune process. A treatment has been associated with improvements in left ventricular ejection fraction, endothelial function, exercise tolerance, and prolonged survival without the need for HTx in DCM patients [196,197,198]. A pilot study involving nine patients with dilated cardiomyopathy and serious heart failure revealed that the removal of IgGs from their plasma via anti-IgG columns resulted in a considerable increase in cardiac functional parameters such as cardiac index and systemic vascular resistance [199]. Research has been conducted to assess the effects of IA followed by the substitution of standard immunoglobulin-G (IA/IgG) in patients with DCM, and the results have been significant, including a rise in cardiac index, left ventricular ejection fraction, and the reduction of symptoms [200,201,202,203]. Results of an observational study demonstrated that patients with dilated cardiomyopathy who received a combination of immunoadsorption and immunoglobulin G substitution experienced positive changes in their New York Heart Association classification, left ventricular ejection fraction, and a 10% reduction in the size of their left ventricular end-diastolic diameter or N-terminal pro-B-type natriuretic peptide [204]. Moreover, IA therapy has been shown to influence the cellular immune response. It suppresses the re-emergence of antibody production in B cells and promotes the expansion of regulatory T cells while reducing the number of activated T cells [205,206]. However, the response to IA/IgG treatment among DCM patients is heterogeneous, with individuals exhibiting diverse patterns of response. Ameling et al. [207] suggested that a combination of cardiodepressant antibody measurement in plasma and gene expression analysis in endomyocardial biopsies of patients with DCM can accurately foresee response to immunoglobulin/immune adsorption therapy. Nevertheless, clinical parameters such as the length of the illness, the performance of the left ventricle, and the presence of myocardial inflammation in endomyocardial biopsies make it impossible to precisely determine the efficacy of immunoglobulin/immune adsorption.

In another study, Bhardwaj et al. [208] aimed to compare the myocardial proteome of responder and non-responder patients with dilated cardiomyopathy (DCM) prior to immunoadsorption (IA) therapy. The results demonstrated that individuals who exhibited a favorable response to the treatment displayed higher levels of Proteins S100-A8, perilipin-4, and kininogen-1 in cardiomyocytes. Additionally, the study identified several differentially abundant proteins involved in immune system function, energy and lipid metabolism, and cardioprotection. Moreover, research has shown that individuals with chronic idiopathic dilated cardiomyopathy (iDCM) and a low level of regulatory T cells (Tregs) are more likely to positively respond to IA therapy [209]. An additional study documented that IA therapy not only eliminates antibodies in DCM, leading to better cardiac function, but also regulates gene expression in DCM. This suggests that IA therapy with immunoglobulin G (IgG) alters the gene expression of desmin in DCM patients, facilitating the clearance of cardiac autoantibodies and alleviating the symptoms of heart failure [210]. Ameling et al. [211] also confirmed that treatment with IA/IgG is associated with a decrease in the gene expression for connective tissue growth factor, fibronectin, and collagen type I in responders, indicating that the enhancement of left ventricular (LV) function following IA/IgG treatment may be attributed to a decrease in the gene expression of heart failure markers and pro-fibrotic molecules, as well as a reduction in fibrosis progression.

In summary, clinical and experimental research have both demonstrated the potential advantages of immunoadsorption (IA) therapy for treating cardiac dysfunction in people with DCM, as it can effectively eliminate various antibodies. However, it is important to note that previous studies have been limited by small sample sizes of DCM and heart failure patients. Therefore, to have a better insight into the lasting outcomes of IA therapy, including mortality and morbidity in DCM patients, further extensive, randomized, prospective, multicenter studies must be conducted.

### 8.4. Cell Therapy

Mesenchymal stem cell (MSC) therapy is considered to have potential benefits for the heart as it activates the natural anti-fibrotic and regenerative responses. Recent meta-analysis has indicated that stem cell therapy can be effective in treating dilated cardiomyopathy, with potential benefits such as an increased left ventricular ejection fraction, a smaller left ventricular end-systolic volume, and a reduced size of the left ventricular end-diastolic chamber [212]. The beneficial effects of MSCs in DCM have been attributed to their ability to increase LVEF, inhibit myocardial fibrosis, improve ventricular remodeling, induce myogenesis and angiogenesis, and enhance exercise tolerance [213,214,215,216]. Furthermore, the extent of improvement in cardiac function following treatment with MSCs may vary with the type of cardiomyopathy. A randomized comparative study revealed that MSC administration had a beneficial effect on cardiac structural and functional parameters in patients with DCM and ischemic cardiomyopathy (ICM) in varying ways. Notably, patients with DCM experienced a considerable improvement in their cardiac systolic function after the MSC therapy, while those with ICM benefited from a positive effect on cardiac remodeling [217]. Mao et al. [218] conducted an animal study and discovered that intramuscular injection of human umbilical cord-derived mesenchymal stem cells (hUCMSCs) could enhance the cardiac function of a doxorubicin-induced dilated cardiomyopathy (DCM) rat model, as well as lessen the mitochondrial and sarcolemma damage related to DCM. Additionally, evidence suggests that the injection of hUCMSCs can boost the myocardial expression and circulating levels of cytokines such as hepatocyte growth factor (HGF), insulin-like growth factor-1 (IGF-1), leukocyte inhibitory factor (LIF), GM-CSF, and VEGF, which implies that the beneficial effects of hUCMSCs on DCM could be attributed to paracrine mechanisms [218]. Zhang et al.’s [219] research uncovered that hUCMSCs, administered in a rat model with myosin-induced dilated cardiomyopathy, could reduce myocardial fibrosis and dysfunction by controlling the expression of TNF-α, ERK1/2, and TGF-β1. This is attributed to the fact that TGF-β1/ERK1/2 signaling plays a significant role in inducing myocardial fibrosis. Furthermore, Studies have shown that hUCMSCs can diminish myocardial fibrosis and EndMT in DCM rats by hindering the activity of TGF-β1/ERK1/2 signaling, suggesting that hUCMSCs could be a potential treatment for DCM [220].

### 8.5. Pharmacological Therapy for DCM

This section summarizes some commonly used heart failure medications approved for the treatment of DCM. Currently, pharmacotherapy for heart failure patients with DCM depends on the patient’s etiology, clinical manifestations, and heart ejection fraction. Majority-type medications, including angiotensin-converting enzyme inhibitors (ACEIs), usually prescribed include captopril, enalapril, fosinopril, lisinopril, perindopril, quinapril, ramipril, and trandolapril. Angiotensin II receptor blockers (ARBs) like candesartan, losartan, valsartan, and β-blockers, lower blood pressure and help strengthen the heart muscle. Sacubitril/valsartan is a new angiotensin-receptor neprilysin inhibitor (ARNI) combination of a neprilysin inhibitor and an ARB [221]. Isosorbide dinitrate, hydralazine, diuretics, and SGLT2 inhibitors also contribute to reducing symptoms of heart failure [PMID: 32749448]. Research has demonstrated the potential of ARNIs as an adjunctive therapy to reduce mortality and hospitalization rates in individuals with DCM, thereby emerging as a potential alternative to ACEIs as a primary treatment option for chronic heart failure [222]. Moreover, the addition of mineralocorticoid antagonists and I_f_ inhibitors to the treatment regimen involving ACEIs and β-blockers has been shown to provide incremental benefits in terms of survival and hospital admissions [221,223]. Notably, findings from the Digitalis Investigation Group trial revealed that while Digoxin did not yield a significant reduction in all-cause mortality, it did exhibit a notable decrease in the relative risk of hospitalization due to heart failure [224].

## 9. Conclusions

DCM is a chronic condition characterized by a complex etiology and high genetic heterogeneity. Recent research from Preclinical studies has revealed the substantial participation of the immune response and its regulatory mechanisms in the emergence and progression of DCM. Gaining a deeper understanding of the crucial roles played by distinct immune cells in DCM development is essential. This understanding will enable us to acquire comprehensive insights into the immune reactions occurring in DCM, thereby identifying more specific immunotherapeutic targets for the treatment of DCM patients. Despite the various immunotherapy approaches available for DCM, debates still exist due to limitations in the existing studies. To make further progress in this field, additional research is necessary to refine current immunotherapy strategies and explore novel immunotherapy alternatives.

## Figures and Tables

**Table 1:** The role of various cells in DCM and related mutant genes.

		Reference
Endothelial cells	Secretes adhesion molecules (P-selectin, E-selectin, ICAM-1, and VCAM-1), which promote the migration of immune cells.	[43,44,45]
Cardiomyocytes	Releasing DAMPs, triggering the production of various chemokines, such as CCL-2, CCL-5, CCL-7, CXCL-1, CXCL-2, and CXCL-8.	[47,48]
Myofibroblasts	Promotes tissue development, wound repair, and fibrosis.	[51,52]
Immune cells		
Monocytes/Macrophages	Secreted cytokines (MCP-1, IL-6, IL-1β, TNF-α, and TGF-β) regulate inflammatory responses and maintain cardiac homeostasis.	[70,71,72,74,82,83]
T cells	● Promotes inflammation and cardiac fibrosis (IL-2, IL-6, IL-1β, TNF, IL-22, and IL-17). ● Secreted anti-inflammatory cytokines (IFN-γ, IL-10.)	[99,101,102,103,104,125]
B cells	● Synthesizing antibodies against cardiac proteins including β1-AR, MHC, and ANT, and others. ● Secreted pro-inflammatory cytokines (TNF-α, lymphotoxin, IL-6, and IL-17), and anti-inflammatory cytokines like IL-10.	[142,143,144,150,151,152]
Natural killer cells	Against acute viral pathogens related to myocarditis and cardiac fibrosis.	[28,174,175]
Neutrophil	Secreting neutrophil gelatinase-related lipocalin and facilitating endothelial cell activation and cytokine release through the formation of neutrophil extracellular traps.	[40,163]
Dendritic cells	As antigen-presenting cells, they transport cellular debris and antigens to T cells.	[30,167]
Genetic mutations	LMNA, TTN, MYH7, PLN, DSP, and SCN5a.	[12,13,14]

Abbreviations: ICAM-1, intracellular cell adhesion molecule-1; VCAM-1, vascular cell adhesion molecule-1; CCL, C-C motif chemokines ligand; CXCL, C-X-C chemokines ligand; MCP-1, monocyte-chemoattractant protein-1; DAMPs, damage-associated molecular patterns; TNF, tumor necrosis factor; IFN, interferon gamma; IL, interleukin; DSP, desmoplakin; LMNA, lamin A/C; MYH7, myosin heavy chain 7; PLN, phospholamban; SCN5a, sodium channel alpha unit 5; TNNT2, troponin T2; TTN, titin; β1-AR, β1 adrenergic receptor; MHC, myosin heavy chain; ANT, adenine nucleotide translocator.

## Data Availability

Not applicable for a review.
